# Revolutionizing renal biopsy: the emerging role of omics and artificial intelligence in nephrology

**DOI:** 10.3389/fmed.2025.1680813

**Published:** 2025-11-04

**Authors:** Nasar Alwahaibi, Maryam Alwahaibi

**Affiliations:** College of Medicine and Health Sciences, Sultan Qaboos University, Muscat, Oman

**Keywords:** renal biopsy, omics techniques, artificial intelligence, deep learning, precision nephrology

## Abstract

Renal biopsies remain indispensable in the diagnosis and management of renal diseases, offering critical histopathological insights that guide clinical decisions. Recent advances in artificial intelligence (AI) and multi-omics technologies have begun to transform renal pathology by enabling deeper molecular profiling, enhanced diagnostic precision, and personalized treatment strategies. Despite these promising developments, challenges such as implementation complexity, cost, and limited integration into routine clinical workflows have slowed widespread adoption. Notably, a significant gap exists in the literature regarding how these modern technologies are applied to maximize the diagnostic and prognostic value of renal biopsies. This mini-review highlights emerging applications of AI and omics in renal biopsy interpretation, emphasizing their potential to transform diagnostic approaches in precision nephrology. It aims to inform nephrologists, renal pathologists, and researchers about the evolving landscape of renal diagnostics, while highlighting areas for further clinical integration and interdisciplinary collaboration.

## Introduction

Renal biopsy remains an essential diagnostic modality in nephrology, offering crucial histopathological insights into various renal diseases such as focal segmental glomerulosclerosis, membranous nephropathy, IgA nephropathy, lupus nephritis, and diabetic nephropathy. It guides disease classification, severity assessment, and therapeutic decision-making, particularly in complex or unclear clinical scenarios (1, 2). While light microscopy, immunofluorescence, and electron microscopy remain the cornerstones of tissue evaluation, the increasing complexity of renal disorders has driven the need for more precise, mechanistic insights beyond histology alone.

Recent advancements in omics technologies, including genomics, transcriptomics, proteomics, and metabolomics, are transforming renal pathology by enabling molecular-level understanding of disease processes. These approaches offer opportunities to identify disease-specific biomarkers, stratify patients by risk, and personalize treatment strategies (3, 4). Similarly, artificial intelligence (AI) is emerging as a powerful tool in renal biopsy analysis, enhancing diagnostic consistency, automating pattern recognition, and integrating histological and molecular data for improved clinical decision-making (5, 6).

Despite these innovations, clinical adoption remains limited, and few studies comprehensively address how omics and AI can be integrated with renal biopsy to enhance diagnostic precision and therapeutic stratification. Current clinical workflows rarely incorporate these tools in a standardized manner, and limited research has addressed their combined utility in enhancing diagnostic precision, monitoring disease progression, or predicting treatment response (7, 8).

This mini-review highlights recent advances in AI and multi-omics integration in renal biopsy interpretation. It focuses on their clinical relevance, diagnostic potential, and translational challenges, aiming to guide nephrologists, renal pathologists, and researchers in adopting modern approaches to kidney disease diagnostics and personalized care.

## Omics technologies

Omics technologies refer to advanced scientific techniques used to study the complete set of molecules within a cell, tissue, or organism. These technologies include genomics (study of genes and DNA), transcriptomics (study of RNA and gene expression), proteomics (study of proteins), and metabolomics (study of metabolites and metabolic pathways), lipidomics (study of lipids), and microbiomics (study of the structure, function, and dynamics of a microbial community) (9). According to the systemic review study, the proteomic approach was the most common ‘omics platform (43.1%), followed by metabolomics (24.4%), genomics (13.8%), epigenomics (8.1%), transcriptomics (4.1) (10). Proteomics techniques include mass spectrometry (MS) combined with ultra-performance liquid chromatography (UPLC) and capillary electrophoresis (CE) (11). Genomics techniques include next generation sequencing (NGS) (12). Metabolomic techniques include high field nuclear magnetic resonance (NMR) and mass spectrometry (MS) coupled with capillary electrophoresis (CE-MS), liquid chromatography MS (LC-MS), or gas chromatography (GC-MS) (13). Transcriptomics techniques include DNA microarrays and RNA sequencing (14). Microbiomics techniques include targeted sequencing of the 16S rRNA gene, and whole metagenome shotgun sequencing (15). Lipidomics techniques include mass spectrometry, liquid chromatography-mass spectrometry, and matrix assisted laser desorption/ionization mass spectrometry imaging (16, 17).

## Clinical applications of omics in renal diseases

Renal biopsy has been revolutionized by multi-omics technologies, which provide a molecular layer of interpretation beyond morphology. By integrating histopathologic features with transcriptomic, proteomic, and metabolomic data, nephrologists can better understand the mechanisms of renal injury, identify early molecular signatures of disease, and discover novel therapeutic targets. Samples from biopsy tissue, urine, and blood can all be analyzed using omics platforms (18).

Omics approaches have enhanced renal biopsy interpretation by revealing how molecular changes correspond to classical histopathologic lesions. For example, transcriptomic studies have demonstrated that reduced intrarenal epidermal growth factor (EGF) expression correlates with tubular atrophy and interstitial fibrosis (TA/IF), refining prognostic assessment within the same morphologic class (19, 20). Urinary proteomic classifiers such as CKD273 detect extracellular matrix remodeling before fibrosis becomes apparent on light microscopy, providing an opportunity for early therapeutic intervention (21). Similarly, multi-omic studies in antineutrophil cytoplasmic antibody (ANCA)–associated vasculitis has shown that proteomic and transcriptomic signatures reflecting neutrophil activation and complement pathways distinguish active inflammation from chronic scarring, even when biopsies appear morphologically similar, improving treatment stratification (22).

Recent work demonstrated that the urinary peptide-based classifier CKD273 can detect early molecular changes preceding morphologic evidence of chronic kidney disease, allowing more precise staging and prognosis beyond what is seen in biopsy alone (23). Likewise, upregulation of retinol dehydrogenase 9 in podocytes was shown to mitigate structural damage, providing mechanistic insight into podocyte injury observed histologically (24). In diabetic kidney disease (DKD), urine metabolomics identified dysregulation of the pantothenate and CoA biosynthesis pathway, linking specific metabolic signatures with characteristic glomerular and tubular lesions (25). Complementing these omics-based insights, a machine-learning model integrating biochemical and clinical data accurately predicted acute kidney injury, demonstrating how computational and omics-driven approaches can refine biopsy interpretation, guide early intervention, and reclassify renal pathologies with greater precision (26).

In lupus nephritis (LN), proximity extension assay proteomics identified urinary ICAM-2, FABP4, FASLG, IGFBP-2, SELE, and TNFSF13B/BAFF as markers distinguishing active LN from inactive SLE, correlating with histologic activity indices (27). In large cohort studies such as the Chronic Renal Insufficiency and Joslin Kidney Studies, integration of proteomic and transcriptomic profiles identified proteins such as TNFRSF1A, FGF20, and ANGPT1 that better predict renal function decline than conventional mesangial-expansion or IFTA scores (28, 29). Another study identified an inflammatory signature of 17 TNF receptor superfamily proteins associated with 10-year end-stage renal disease risk, supporting their potential use as biomarkers and therapeutic targets (30).

Collectively, these examples illustrate that omics does not replace histopathology but rather augments it, transforming static microscopic patterns into dynamic molecular phenotypes that improve diagnostic precision, prognostic accuracy, and personalized patient management. The integration of omics data with renal biopsy thus represents a paradigm shift in nephrology, offering a comprehensive view that bridges morphology with molecular mechanisms. However, it remains important to recognize that these technologies are still evolving and require further validation before their routine clinical adoption.

## Omics technologies: challenges and solutions

While omics technologies offer transformative potential in renal biopsy analysis, their clinical application faces several challenges. One major limitation is the high cost, complexity of omics techniques, and large sample size is required, which can limit their accessibility in resource-limited settings (31, 32). To address the challenges, solutions include reducing costs through improved technology and automation, focusing on targeted biomarker panels, using machine learning to analyze smaller datasets, fostering collaborations for shared data, developing portable and cost-effective platforms, and optimizing sample preparation and data analysis protocols (31). These approaches can make omics more accessible and applicable in clinical settings, even in resource-limited environments. In addition, the massive amount of data generated by omics analyses requires advanced bioinformatics tools and expertise for interpretation, which may not be readily available in all clinical laboratories (33, 34). To address these challenges, collaborative efforts between researchers, clinicians, and bioinformaticians are essential to develop cost-effective and user-friendly omics platforms (35). Another issue is the lack of standardized protocols for sample preparation, data analysis, and interpretation, which can lead to variability in results. Establishing standardized guidelines and quality control measures can help improve the reproducibility and reliability of omics-based diagnostics (36).

## AI in renal biopsy

In recent years, the change from human systems to machine systems [artificial intelligence (AI)], has been a great progress in the field of medical imaging, including renal pathology (37). AI techniques are increasingly being integrated into renal biopsy analysis, significantly enhancing diagnostic accuracy and efficiency. Machine learning algorithms, particularly deep learning, can be trained to identify and classify various renal diseases by analyzing histopathological images with high precision. This prognostic study found better performance for quantifying percent global glomerulosclerosis from whole-slide images of frozen and of permanent hematoxylin-eosin–stained donor transplant kidney biopsy specimens by a deep learning model (94% accuracy) than by on-call board-certified pathologists (80%) (38). Similarly, Convolutional neural networks (CNNs)-based systems indicated that this technique is suitable for correct glomerulus detection in Whole Slide Images, showing robustness while reducing false positive and false negative detections (39). These findings underscore the importance of hybrid approaches combining AI with expert histopathological evaluation.

These AI models can detect subtle changes in tissue structure, cellular patterns, and disease markers that might be overlooked by human eyes, leading to earlier and more accurate diagnoses. AI can also assist in quantifying the extent of fibrosis, inflammation, and other pathological features, providing standardized and reproducible assessments (40). Moreover, AI-driven image analysis can streamline the workflow of pathologists, allowing them to focus on complex cases and improving overall diagnostic throughput (41).

Various AI techniques are used for the analysis of renal biopsy samples, enhancing the accuracy and efficiency for diagnoses. CNNs, a type of deep learning, are effective in analyzing histopathological images (42). CNN and its variants are the most common neural networks utilized for categorizing renal carcinoma pathology images (43). CNNs can automatically identify and classify different renal diseases by learning from large datasets of annotated biopsy images, detecting patterns and features that may be imperceptible to human pathologists (44, 45). Another AI technique, machine learning algorithms like support vector machines (SVMs) and random forests, can be employed to analyze quantitative features extracted from biopsy images, such as glomerular size, tubular atrophy, and interstitial fibrosis (46). These algorithms can classify disease severity and predict outcomes based on the extracted features. In addition, natural language processing (NLP) can be used to analyze pathology reports and integrate clinical data with histopathological findings, providing a comprehensive diagnostic approach (47). Joint learning, which combines multiple AI models to improve prediction accuracy, is also used to enhance the robustness of biopsy analysis. These AI techniques not only improve diagnostic accuracy and reproducibility but also facilitate personalized treatment plans and better patient management in nephrology (48).

## Clinical applications of AI in renal diseases

AI has demonstrated significant potential in improving the diagnosis and management of kidney diseases. For example, in a clinical study involving 948 patients with IgA nephropathy (IgAN), artificial neural networks (ANNs) successfully identified individuals at high risk of developing end-stage kidney disease (ESKD) and predicted the time-to-event endpoint, aiding in risk assessment and early intervention (49). Another study focused on predicting renal flare in 1,694 patients with biopsy-proven LN and stratifying risk to enhance clinical decision-making and personalized management. The XGBoost model and the simplified risk score prediction model (SRSPM) effectively predicted renal flare in LN, with SRSPM also enabling risk stratification, ultimately supporting improved kidney outcomes (50). Additionally, a study aiming to enhance kidney disease severity assessment beyond traditional semiquantitative scoring utilized image digitization and morphometric techniques on 300 biopsy samples. The results showed that six CNN models outperformed pathologists in estimating the percentage of interstitial fibrosis, demonstrating the potential for AI in histopathological evaluation (51).

**FIGURE 1:**
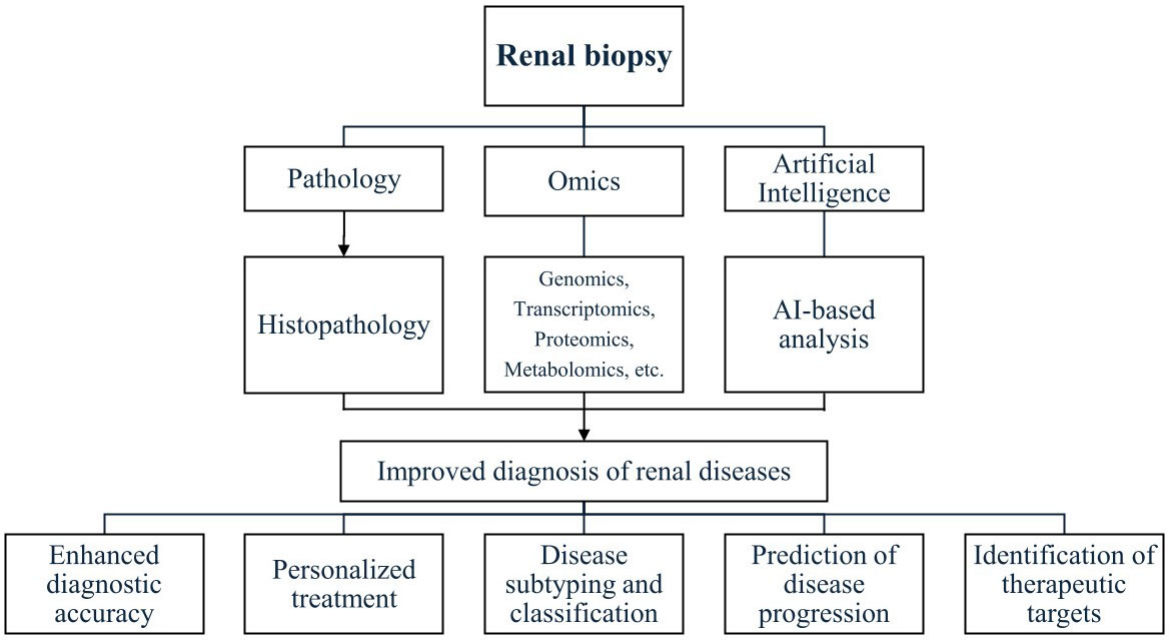
Integration of traditional renal biopsy assessment with omics technologies and artificial intelligence to enhance diagnostic accuracy.

Another study developed a deep learning model for continuous risk prediction of patient deterioration, using acute kidney injury as an example. Trained on electronic health records from 703,782 adult patients across diverse clinical settings, the model predicted 55.8% of all inpatient AKI cases and 90.2% of severe cases requiring dialysis, with a lead time of up to 48 h. It also provided confidence assessments, highlighted key clinical features, and predicted blood test trajectories, offering a valuable tool for early intervention and improved patient outcomes (52). Furthermore, an artificial neural network model was developed to predict ESKD in patients with primary IgAN using a retrospective cohort of 948 patients. The model included a classifier for ESKD prediction and a regression model for estimating time to onset. Performance improved over time, achieving an area under the curve of 0.82 at 5 years and 0.89 at 10 years. External validation in 167 patients showed successful predictions for 91%, with superior discrimination (Harrell C index: 81% at 5 years, 86% at 10 years) and calibration compared to other models. The tool demonstrated strong predictive accuracy over a 25-year follow-up period, effectively identifying high-risk patients and supporting early therapeutic strategies to enhance clinical outcomes (49). Figure 1 shows an integration of traditional renal biopsy assessment with omics technologies and artificial intelligence to enhance diagnostic accuracy.

## AI in renal biopsy: challenges and solutions

The integration of AI into renal biopsy analysis also presents several challenges. One significant limitation is the need for large, high-quality datasets to train AI models effectively. Inadequate or biased datasets can lead to inaccurate predictions and reduced generalizability of AI algorithms (53). To overcome this, multicenter collaborations and data-sharing initiatives are critical to build strong and diverse datasets. Another challenge is the black box nature of some AI algorithms, where the decision-making process is not transparent, making it difficult for clinicians to trust and interpret AI-generated results (54, 55). Developing explainable AI models and providing training for pathologists on AI tools can help bridge this gap. In addition, the integration of AI into clinical workflows requires significant infrastructure and training, which may pose logistical and financial challenges for healthcare institutions (56). Gradually introducing AI technologies and continuously training healthcare providers can help make the transition easier. Another significant issue is related to ethical and safety concerns. Current laws and regulations are insufficient to address issues surrounding patient privacy, data security, and data ownership (57). To overcome these challenges, it is crucial to establish an international consensus on the ethical and safe use of AI in renal pathology, ensuring that these concerns are addressed and managed effectively by the global community (58).

## Conclusion

The integration of advanced technologies such as omics and AI into renal biopsy interpretation represents a transformative shift in nephrology. These tools enable deeper molecular insights, enhance diagnostic accuracy, and support the development of personalized treatment strategies. Omics technologies provide a comprehensive understanding of the complex biological pathways underlying renal diseases, while AI offers automation, standardization, and predictive power in image and data analysis. Together, they hold the potential to improve patient outcomes and drive precision medicine in nephrology. Despite their promise, challenges remain in terms of clinical implementation, data interpretation, infrastructure, and ethical considerations. Addressing these issues through standardization, interdisciplinary collaboration, and robust validation studies is essential for successful clinical integration. As research continues to advance, the role of AI, alongside omics, in renal biopsy is expected to become increasingly crucial. Embracing these innovations will be key to enhancing diagnostic precision and improving patient care in the era of precision nephrology.
